# Body fat distribution and risk of incident ischemic stroke in men and women aged 50 to 74 years from the general population. The KORA Augsburg cohort study

**DOI:** 10.1371/journal.pone.0191630

**Published:** 2018-02-05

**Authors:** Karl Zahn, Jakob Linseisen, Margit Heier, Annette Peters, Barbara Thorand, Franziska Nairz, Christa Meisinger

**Affiliations:** 1 Helmholtz Zentrum München, German Research Center for Environmental Health, Institute of Epidemiology II, Neuherberg, Germany; 2 Institute for Medical Informatics, Biometry and Epidemiology (IBE), Munich, Germany; 3 Chair of Epidemiology, Ludwig-Maximilians-Universität München, UNIKA-T, Augsburg, Germany; Yale University School of Medicine, UNITED STATES

## Abstract

**Background:**

It remains controversial whether measures of general or abdominal adiposity are better risk predictors for ischemic stroke. Furthermore, so far it is unclear whether body fat mass index (BFMI) and fat free mass index (FFMI) are risk predictors for ischemic stroke. This study examined the sex-specific relevance of body mass index (BMI), BROCA Index, waist circumference (WC), waist-height ratio (WHtR), BFMI and FFMI for the development of ischemic stroke in a Caucasian population.

**Material and methods:**

The prospective population-based cohort study was based on 1917 men and 1832 women (aged 50 to 74 years) who participated in the third (1994/95) or fourth (1999/2001) MONICA/KORA Augsburg survey. Subjects were free of stroke at baseline. Standardized anthropometric and bioelectric impedance measurements were obtained at baseline. Hazard ratios (HR) were estimated from Cox proportional hazard models.

**Results:**

During a median follow-up of 9.3 years 128 ischemic strokes occurred in men and 81 in women, respectively. Coded as quartiles WC and WHtR were significantly associated with incident stroke in multivariable analyses in women (comparing the 4^th^ vs. the bottom quartile), but none of the adiposity measures was significantly associated with incident stroke in multivariable adjusted analyses in men. When anthropometric measures were used as continuous variables, these findings were confirmed. After multivariable adjustment the associations between obesity measures and incident ischemic stroke were statistically significant only for WC (HR 1.39, 95%CI 1.12-1.72) and WHtR in women (HR 1.39, 95%CI 1.12-1.73) per increase of 1 standard deviation. In both sexes the measures BFMI and FFMI were no independent predictors for incident ischemic stroke.

**Conclusions:**

Abdominal obesity measures are independent predictors of incident ischemic stroke in women but not in men from the general adult population. Thus, it may be of particular importance for women to prevent central obesity in order to reduce their risk of ischemic stroke.

## Introduction

Ischemic stroke has become one of the leading causes of disability and mortality worldwide [1]. The etiology of ischemic stroke is not well understood, but it is known that a large number of biological and lifestyle factors, such as hypertension, diabetes mellitus, alcohol consumption, smoking [2], and also genetic factors [3] are involved in the development of the disease. Literature search and meta-analyses confirm that a large number of prior longitudinal studies assess overweight and obesity as relevant risk factors for ischemic stroke. Some articles concentrate on obesity defined by a body mass index (BMI) of ≥ 30 kg/m^2^ [4–6]. However, BMI as a measure of general obesity does not take into account body fat distribution. Thus it is not possible to distinguish between persons with excess adipose tissue and those with high muscle mass. Consequently, incorrect risk estimates associated with obesity will result for persons with high muscle mass when only BMI is considered. Therefore, recent studies focused on abdominal obesity measures when investigating the obesity-related risk for ischemic stroke [7–9]. They found that abdominal fat measures like waist circumference (WC) or waist-height ratio (WHtR) are better risk predictors for ischemic stroke than BMI only [7,8]. It is well known that body fat distribution is sex-specific. Men tend to accumulate adipose tissue in the abdominal region, and women tend to accumulate it around the hip [10]. However, menopausal women after the fifties also tend to accumulate abdominal adipose tissue [11]. Abdominal (visceral) fat is endocrine-active and releases inflammatory markers, which are of importance in the pathogenesis of cardiovascular disease (CVD) [12] and there are sex-differences regarding the association between markers of inflammation and CVD risk [13]. Contrary, peripheral obesity with adipose deposition around the hips seems to have protective effects on CVD risk [14].

While prior investigated general and abdominal obesity measures are often height dependent (e.g. BMI, WHtR) the use of body fat mass and fat free mass allows a height-independent evaluation of the association between body fat and stroke risk [15]. Until now there are only a few studies dealing with body fat mass and CVD [16,17] and so far, there are no population-based studies on middle-aged men and women evaluating the influence of body fat mass and fat free mass on the development of incident ischemic stroke.

The aim of this study was therefore to assess sex-specific associations between general and abdominal obesity measures and incident ischemic stroke, using a population-representative sample of 1917 men and 1832 women aged 50-74 years without a history of stroke. In addition, it was investigated, whether body fat mass index (BFMI) and fat free mass index (FFMI) are independent predictors for incident ischemic stroke.

## Material and methods

### Study population

The present analyses were performed using data from the prospective, population-based cohort study, Monitoring Trends and Determinants in Cardiovascular Diseases (MONICA)/ Cooperative Health Research in the Region of Augsburg (KORA). Details of the study have been described previously [18, 19,20].

**Fig 1:**
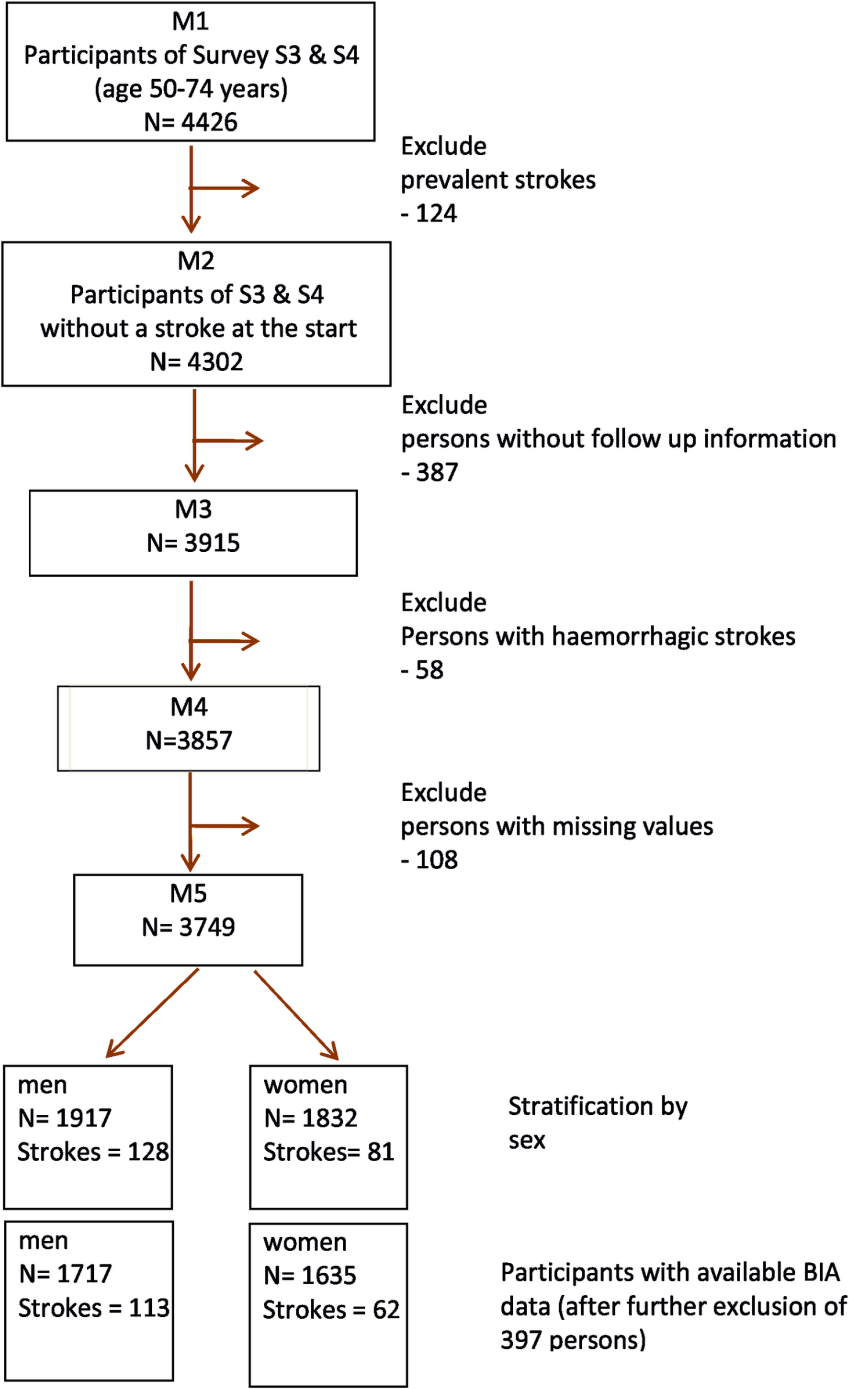
Flow chart for the inclusion of the study participants.

The data source used in the present analysis was the third (S3) and fourth (S4) cross-sectional survey of the MONICA/KORA Augsburg project conducted in 1994/95 and 1999/2001, respectively. The study participants comprised 4856 (S3) and 4261 (S4) men and women (response 75% and 68%, respectively). Because there are only a few ischemic stroke events below the age of 50, the initial sample included 4426 persons aged 50–74 years. After exclusion of participants with prevalent stroke (n = 124), persons without follow-up information (n = 387), and participants with haemorrhagic stroke (n = 58) there remained n = 3857 participants (1972 men, 1885 women). All men and women with missing data on any of the considered risk factors were also excluded (*n* = 108). Finally, 3749 study participants (1917 men and 1832 women) aged 50–74 years formed the basis of this report. Because body impedance analysis was conducted in a part of the participants of S4 only, the analysis regarding the association between BFMI and FFMI and incident ischemic stroke was based on 3352 participants (1717 men and 1635 women) (Fig 1).

The study was approved by the ethics committee of the Bavarian Medical Association (“Bayerische Landesärztekammer”) and performed in accordance with the Declaration of Helsinki. All study participants gave their written informed consent to take part in the study.

### Outcome variable

The study endpoint was incident ischemic (non-fatal plus fatal) strokes/transient ischemic attacks (TIA) during the follow-up period (from the time point of the baseline examination until the end of the follow-up on December 31, 2009). Non-fatal strokes were assessed by postal follow-up questionnaires. Using data from participants’ hospital records and information gathered from their attending physicians, all self-reported potential incident stroke cases and the date of diagnosis were validated. To ascertain fatal strokes, the survival status of all participants was regularly checked using information provided by population registries inside and outside the study area. Death certificates of deceased participants, preserved by local health authorities, were analysed and the main causes of death were extracted. As mentioned above, all information regarding fatal strokes was also checked by interviewing the participant’s last attending physician, as well as by screening his hospital records. Additionally, the same procedure was used to examine if an individual who had died from a cause other than a stroke, had suffered a stroke in the time between the last follow-up and the participant`s eventual death. The following three-digit International Classification of Diseases, Ninth Revision (ICD-9) German modification codes were considered as death due to stroke: 433, 434, and 435.

### Measurement of exposure

Exposures are the different obesity and body composition measures. Data on height, weight, waist circumference (WC) and body impedance measurement were collected in a standardized physical examination at baseline. Anthropometric measurements were taken after participants had removed shoes, heavy clothing, belts and corsets. Body weight was measured in light clothing to the nearest 0.1 kg and height to the nearest 0.5 cm. Waist circumference was measured at the level midway between the lower rib margin and the iliac crest with the participant breathing out gently. Standing height was measured with a fixed stadiometer calibrated in centimetre, body weight in kilogram by using balance-beam scales. For the assessment of body composition, two bioelectrical impedance analysis measurements of resistance (R), reactance (Xc) and the phase angle (α) were taken between the dominant hand wrist and dorsum and the dominant foot angle and dorsum (placement of the electrodes) by means of a body impedance analyser (BIA 2000-S; Data Input GmbH, Frankfurt, Germany) while the non-fasting subjects were spreading their arms and legs and lying in a relaxed and supine position on a nonconductive surface with 50 kHz [21]. Fat free mass, and fat mass were then calculated by means of Kyle’s equations on which the following indices are based: FFMI (fat free mass in kg/(height in m)^2^), body fat mass index (body fat mass in kg/(height in m)^2^). [22].

Body mass index (BMI) was calculated as the ratio of weight in kilogram to height in meter squared. Furthermore, the BROCA Index (BROCA) was used:

$$BROCA_{normal}=height(cm)-100$$

$$BROCA=\frac{weight(kg)-{BROCA}_{normal}}{{BROCA}_{normal}}*100\%$$

Waist-height ratio (WHtR) was calculated as WC divided by height. Body fat mass index (BFMI) was calculated by body fat mass divided by height squared; fat free mass index (FFMI) by fat free mass divided by height squared.

### Baseline information and measurement of the covariates

Each participant took part in a face-to-face interview by trained staff in the baseline surveys. Demographic information, data on socioeconomic factors, lifestyle habits (tobacco use and alcohol consumption) and health history (e.g. self-reported or physician diagnosed diabetes, myocardial infarction) were gathered. The educational level of a participant was estimated by years of education; ≤10 years being classified as low educational level, 11-12 years as middle, and ≥13 years as high educational level. Participants were classified as active during leisure time if they regularly participated in sports in the summer and winter and if they were active for ≥1 hour/week in either season. Participants were classified into current cigarette smokers (occasionally or regularly), ex-smokers and never-smokers.

Actual hypertension was defined as blood pressure ≥140/90 mmHg and/or the consumption of antihypertensive medication, given that the subjects were aware of being hypertensive. Blood pressure was measured with a random-zero sphygmomanometer in S3 (Hawksley & Sons Ltd, Lancing, England) and by use of an oscillometric digital blood pressure monitor (HEM-705CP, Omron Corporation, Tokyo, Japan) in S4 in a sitting position. Recently, a calibration study was conducted to compare the different devices [23]. Drugs, which had been consumed during the past 7 days before study examination, were recorded and classified by agents following the anatomic-therapeutic-chemical classification system [24].

A non-fasting blood sample was collected while sitting. Methods used to measure total cholesterol and HDL cholesterol are expounded in detail elsewhere [19,20].

### Statistical analysis

Baseline characteristics were calculated separately for men and women with and without incident stroke. For dichotomous variables proportions and for continuous variables means ± 1 standard deviation (SD) were reported. If a normal distribution could be assumed the t-test was applied for comparison of the means, else the Wilcoxon test for comparison of the medians was used. The Chi square-test was used to compare proportions.

Pearson’s correlation coefficients were calculated to assess the relationship between the different anthropometric measures.

Non parametric survival analysis for gender was carried out with Kaplan Meier methods. Further analyses were performed separately for men and women. Cox proportional hazard models were used to calculate the hazard ratios (HR) and 95% confidence intervals (95% CI) by quartiles (reference category first quartile) of the different obesity measures. To investigate in addition, whether the persons at the top 10% of a measure had an extremely high risk for ischemic stroke, the RR for the ≥90^th^ percentile (reference category ≤25^th^ percentile) was calculated. For the analysis of the obesity measures as continuous variables the HR for a 1-standard deviation increase in the measure was regarded.

Models were built for groups of confounding factors: Model 1 was adjusted for age and survey, Model 2 additionally for family history of stroke, physical activity, educational level, alcohol consumption and smoking, and Model 3 additionally for history of diabetes and hypertension. The assumption of proportional hazard was tested graphically by Schoenlein-Hennoch residuals. The fits of the models were compared using the likelihood ratio test.

Statistical analyses were performed with R-program [25] and libraries and statistical significance was defined as *P*<0.05.

## Results

**Fig 2:**
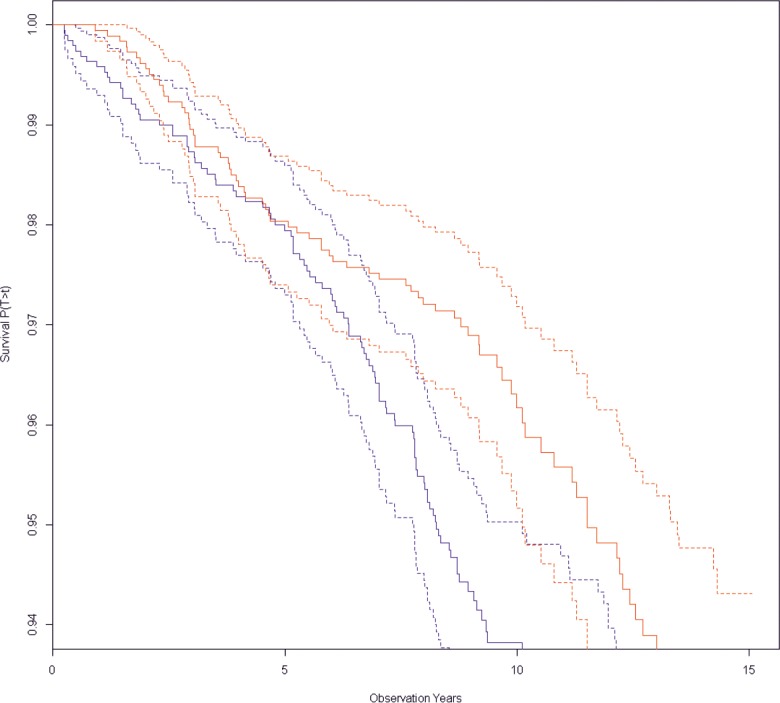
Kaplan Meier survival curves for women (n = 1832, in red, at the top) and men (n = 1917, in blue, at the bottom). Dashed lines are 95% CI. Factor is sex, outcome is incident stroke, logrank test 10.8 on 1 df, p<0.00099.

The study included 1917 men and 1832 women. A 'first ever stroke' appeared in 128 men and 81 women. The median observation time (until stroke or censoring) was 9.3 years with ranges between 39 days and 15.1 years. The cumulative incidence rates for ischemic stroke in men were 68.7/10,000 observation years and in women 43.6/10,000 observation years. Kaplan-Meier survival curves for women and men were drawn (Fig 2), they were significantly different (logrank test 10.8 on 1 degree of freedom (df), p = 0.00099).

Table 1 presents the sex-specific baseline characteristics for persons with and without incident ischemic stroke during follow-up. At baseline, the median age was about 60 years in men and women without an incident stroke and 64 years in men and 65 years in women with stroke during follow-up.

**Table 1:** Baseline characteristics of the study participants with and without incident ischemic stroke during follow-up. Data are given as mean values and standard deviations (SD) and percentages if not otherwise mentioned.

Variables	sex	No_stroke_event	Incident_stroke	p_values
**Demographic factors**				
**Sex**	total	3540	209	
	men	1789 (50.5%)	128 (61.2%)	
	women	1751 (49.5%)	81 (38.8%)	
**Age[years], median (min-max)**	men	60 (50-74)	64 (50-74)	0.0001
	women	60 (50-74)	65 (51-74)	<0.0001
**Observation time[years], med (min-max)**	men	9.2 (0.1-15)	7.0 (0.3-14.6)	
	women	9.3 (0.3-15.1)	6 (0.9-14.3)	
**Obesity measures**				
**Height[cm], mean (sd)**	men	172.18 (6.31)	170.91 (6.37)	0.031
	women	159.57 (6.01)	158.3 (5.81)	0.058
**Weight[cm], mean (sd)**	men	83.62 (11.89)	84.35 (13.08)	0.54
	women	72.08 (12.60)	74.46 (13.98)	0.137
**BROCA[%], mean (sd)**	men	16.12 (15.45)	19.10 (16.26)	0.046
	women	21.70 (21.68)	28.01 (22.07)	0.014
**BMI[kg/m^2], mean (sd)**	men	28.19 (3.68)	28.83 (3.91)	0.075
	women	28.33 (4.86)	29.67 (5.14)	0.024
**WC[cm], mean (sd)**	men	99.73 (9.67)	101.63 (10.49)	0.048
	women	88.69 (11.43)	94.21 (11.61)	<0.0001
**WHtR, mean (sd)**	men	0.58 (0.06)	0.59 (0.06)	0.007
	women	0.56 (0.08)	0.60 (0.07)	<0.0001
BFMI[kg/m^2], mean (sd)*	men	8.26 (2.27)	8.77 (2.54)	0.041
	women	11.18 (3.18)	12.28 (3.65)	0.022
FFMI[kg/m^2], mean (sd)*	men	19.96 (1.77)	20.19 (1.93)	0.208
	women	17.11 (1.87)	17.51 (1.90)	0.108
**Covariates**				
**HDL[mg/dl], mean (sd)**	men	49.99 (14.31)	48.98 (14.15)	0.435
	women	61.12 (17.08)	57.46 (19.52)	0.101
**Cholesterol[mg/dl], mean (sd)**	men	237.83 (43.17)	239.84 (40.27)	0.59
	women	246.49 (41.39)	254.33 (37.01)	0.067
**Alcohol-Intake, median (Q1, Q3)**	men	20 (3.7, 40)	20 (5.7, 40)	0.939
	women	2.86 (0, 10.7)	0 (0, 14.3)	0.455
**Hypertension[%]**	men	56.9%	76.6%	<0.0001
	women	52%	60.5%	0.165
**History of diabetes[%]**	men	6.7%	13.3%	0.009
	women	5.5%	18.5%	<0.0001
**Family history of stroke[%]**	men	26.3%	31.2%	0.259
	women	30.4%	40.7%	0.066
**Low level of physical activity[%]**	men	57.8%	61.7%	0.438
	women	59.4%	71.6%	0.038
**Smoker (Yes, Ex, Never)**				
**Yes[%]**	men	20.2%	24.2%	0.115
**Ex[%]**	men	48.5%	53.1%	
**Yes[%]**	women	12.6%	13.6%	0.951
**Ex[%]**	women	19.2%	19.8%	
**Education(low, mid,high)**				
**low[%]**	men	55.2%	62.5%	0.278
**mid[%]**	men	21.3%	18%	
**low[%]**	women	74.1%	87.7%	0.022
**mid[%]**	women	14.4%	7.4%	

*based on 1717 men and 1635 women

In both sexes, the obesity measures BROCA, WC, WHtR and BFMI were significantly higher in persons who developed a first ever stroke in comparison to persons without a stroke event during follow-up. Only women with an incident stroke at follow-up had a significantly higher BMI in comparison to women without stroke. Men who suffered from a stroke were smaller than men without a stroke.

Women who developed a stroke had more often a lower education and were less often physically active. Persons who developed a stroke during follow-up reported more often a history of diabetes, had higher blood pressure (men only) and higher total cholesterol values (women only). Table 2 shows the distribution of the obesity measures separately for men and women. We calculated the correlation coefficients between obesity measures BMI, BROCA, WC, WHtR, and BFMI and observed high correlation coefficients: R = 0.87 for WC-BMI in men and R = 0.97 for BFMI-BMI in women (Table 3). Tests for interactions between the obesity measures as categorical or continuous variables and sex, age, and survey were not significant in the corresponding models.

**Table 2:** Cut-off values for height, weight and the obesity measures (in addition mean and SD).

measure	sex	Minimum	First Q	Median	Mean	Third Q	Maximum	SD
height[cm]	men	153	168	172	172.1	176.4	194.5	6.3
	women	138.7	155.5	159.5	159.5	163.5	179	6
weight[kg]	men	44.3	75.4	83	83.7	91	143	12
	women	43.8	63	70.7	72.2	79.6	142	12.7
BMI[kg/m^2]	men	16.2	25.8	27.8	28.2	30.3	46.3	3.7
	women	17.8	25	27.8	28.4	31.1	51.2	4.9
BROCA[%]	men	-32.1	5.6	14.6	16.3	25.1	90.1	15.5
	women	-25.9	6.4	19.2	22	34	118.5	21.7
WC[cm]	men	70.2	93.4	99	99.8	105	140.5	9.7
	women	64	80.7	87.9	88.9	96.1	137.2	11.5
WHtR	men	0.4	0.5	0.6	0.6	0.6	0.8	0.1
	women	0.4	0.5	0.6	0.6	0.6	0.8	0.1
BFKG[kg]	men	5	19.9	24	24.6	28.8	59.3	6.9
	women	7.1	22.6	27.8	28.5	33	69.7	8.1
BFMI[kg/m^2]*	men	1.8	6.8	8.1	8.3	9.5	18.4	2.3
	women	3.4	8.9	10.9	11.2	13.1	25.3	3.2
FFMI[kg/m^2]*	men	13.8	18.8	19.8	20	20.9	28.8	1.8
	women	12.8	15.8	16.9	17.1	18.2	27.4	3.2

*based on 1717 men and 1635 women

**Table 3:** Pearson correlation coefficients between the different obesity measures (right and up men).

	Age	Height	Weight	BMI	WC	WHtR	BFMI*	FFMI*
**Age**	1	-0,25	-0,09	0,04	0,10	0,19	0,13	-0,07
**Height**	-0,31	1	0,42	-0,1	0,10	-0,27	-0,04	-0,16
**Weight**	-0,01	0,26	1	0,86	0,85	0,66	0,82	0,72
**BMI**	0,13	-0,17	0,91	1	0,87	0,88	0,93	0,88
**WC**	0,19	-0,01	0,85	0,87	1	0,93	0,90	0,66
**WHtR**	0,27	-0,29	0,74	0,88	0,96	1	0,89	0,70
BFMI*	0,16	-0,13	0,89	0,97	0,88	0,88	1	0,65
FFMI*	0,03	-0,19	0,81	0,92	0,76	0,78	0,8	1

*based on 1717 men and 1635 women

Table 4 presents the HRs of first ever stroke for the obesity measures in quartiles and separately for the top 10% (≥90. percentile) - each category with the bottom quartile as reference.

**Table 4:** Sex-specific RR of stroke for the different obesity measures by quartiles and top 10% (≥90^th^ percentiles); reference category: Bottom quartile.

.	First quartile	Second quartile	Third quartile	Fourth quartile	top10
	-	-	-	-	-
**MEN**					
**BMI**					
**Strokes (N)**	27	31	31	39	18
**Model 1**	1.00	1.07 (0.64-1.80)	1.03 (0.61-1.72)	1.42 (0.87-2.32)	1.80 (0.99-3.28)
**Model 2**	1.00	1.09 (0.65-1.84)	1.04 (0.62-1.75)	1.40 (0.85-2.30)	1.76 (0.96-3.23)
**Model 3**	1.00	1.02 (0.61-1.72)	0.88 (0.52-1.50)	1.15 (0.70-1.91)	1.43 (0.78-2.65)
**BROCA**					
**Strokes (N)**	25	30	32	41	19
**Model 1**	1.00	1.14 (0.67-1.94)	1.09 (0.65-1.85)	1.62 (0.98-2.66)	1.98 (1.09-3.59)
**Model 2**	1.00	1.16 (0.68-1.99)	1.11 (0.65-1.88)	1.60 (0.97-2.65)	1.97 (1.07-3.63)
**Model 3**	1.00	1.11 (0.65-1.90)	0.94 (0.55-1.60)	1.32 (0.79-2.21)	1.62 (0.87-3.00)
**WC**					
**Strokes (N)**	26	27	40	35	19
**Model 1**	1.00	0.99 (0.58-1.70)	1.61 (0.98-2.64)	1.47 (0.88-2.44)	2.08 (1.15-3.78)
**Model 2**	1.00	0.99 (0.58-1.70)	1.60 (0.97-2.63)	1.38 (0.83-2.31)	1.97 (1.08-3.59)
**Model 3**	1.00	0.94 (0.54-1.61)	1.38 (0.84-2.29)	1.18 (0.70-1.98)	1.57 (0.85-2.88)
**WHtR**					
**Strokes (N)**	25	23	37	43	18
**Model 1**	1.00	0.87 (0.49-1.54)	1.41 (0.85-2.36)	1.72 (1.04-2.85)	1.92 (1.04-3.56)
**Model 2**	1.00	0.82 (0.46-1.45)	1.36 (0.81-2.28)	1.62 (0.97-2.70)	1.79 (0.96-3.35)
**Model 3**	1.00	0.75 (0.42-1.34)	1.14 (0.67-1.92)	1.31 (0.78-2.20)	1.41 (0.74-2.66)
BFMI*					
**Strokes (N)**	22	28	29	34	15
**Model 1**	1.00	1.15 (0.66-2.01)	1.19 (0.68-2.07)	1.57 (0.91-2.7)	1.88 (0.97-3.64)
**Model 2**	1.00	1.13 (0.64-1.98)	1.20 (0.68-2.10)	1.47 (0.85-2.54)	1.75 (0.90-3.40)
**Model 3**	1.00	1.02 (0.58-1.81)	1.04 (0.59-1.83)	1.17 (0.67-2.05)	1.40 (0.71-2.74)
FFMI*					
**Strokes (N)**	26	26	25	36	17
**Model 1**	1.00	0.92 (0.53-1.58)	0.92 (0.53-1.59)	1.38 (0.83-2.28)	1.79 (0.97-3.31)
**Model 2**	1.00	0.96 (0.55-1.65)	0.91 (0.52-1.58)	1.39 (0.83-2.31)	1.82 (0.98-3.39)
**Model 3**	1.00	0.91 (0.53-1.58)	0.86 (0.49-1.49)	1.19 (0.71-1.99)	1.53 (0.82-2.87)
**WOMEN**	-	-	-	-	-
**BMI**					
**Strokes (N)**	14	17	26	24	14
**Model 1**	1.00	1.11 (0.55-2.25)	1.57 (0.82-3.01)	1.50 (0.77-2.91)	2.23 (1.06-4.70)
**Model 2**	1.00	1.12 (0.55-2.29)	1.55 (0.80-3.01)	1.51 (0.77-2.97)	2.13 (1.00-4.54)
**Model 3**	1.00	1.16 (0.57-2.37)	1.58 (0.81-3.09)	1.35 (0.67-2.70)	1.82 (0.82-4.01)
**BROCA**					
**Strokes (N)**	12	20	25	24	13
**Model 1**	1.00	1.41 (0.69-2.89)	1.7 (0.85-3.41)	1.63 (0.81-3.28)	2.24 (1.02-4.94)
**Model 2**	1.00	1.44 (0.70-2.96)	1.71 (0.84-3.45)	1.65 (0.81-3.37)	2.15 (0.96-4.80)
**Model 3**	1.00	1.40 (0.67-2.90)	1.72 (0.85-3.51)	1.47 (0.70-3.07)	1.80 (0.77-4.16)
**WC**					
**Strokes (N)**	8	18	24	31	16
**Model 1**	1.00	1.97 (0.85-4.54)	2.55 (1.14-5.70)	3.47 (1.58-7.62)	4.79 (2.03-11.28)
**Model 2**	1.00	1.91 (0.82-4.42)	2.54 (1.13-5.71)	3.39 (1.53-7.54)	4.66 (1.95-11.17)
**Model 3**	1.00	1.96 (0.84-4.56)	2.57 (1.14-5.81)	3.26 (1.44-7.35)	3.97 (1.62-9.71)
**WHtR**					
**Strokes (N)**	9	12	28	32	16
**Model 1**	1.00	1.19 (0.50-2.84)	2.44 (1.14-5.22)	2.97 (1.40-6.32)	3.76 (1.64-8.65)
**Model 2**	1.00	1.18 (0.49-2.82)	2.38 (1.11-5.13)	2.97 (1.37-6.42)	3.74 (1.59-8.85)
**Model 3**	1.00	1.22 (0.51-2.93)	2.52 (1.16-5.47)	2.82 (1.28-6.19)	3.26 (1.34-7.91)
BFMI*					
**Strokes (N)**	12	12	16	22	13
**Model 1**	1.00	0.82 (0.37-1.83)	1.11 (0.52-2.35)	1.45 (0.71-2.96)	2.43 (1.10-5.34)
**Model 2**	1.00	0.83 (0.37-1.86)	1.14 (0.53-2.45)	1.36 (0.66-2.80)	2.29 (1.01-5.18)
**Model 3**	1.00	0.86 (0.38-1.94)	1.18 (0.55-2.55)	1.21 (0.58-2.54)	2.00 (0.87-4.61)
FFMI*					
**Strokes (N)**	11	15	20	16	10
**Model 1**	1.00	1.42 (0.65-3.09)	1.69 (0.81-3.53)	1.52 (0.7-3.28)	2.60 (1.10-6.12)
**Model 2**	1.00	1.34 (0.61-2.93)	1.73 (0.82-3.65)	1.50 (0.69-3.28)	2.48 (1.03-5.95)
**Model 3**	1.00	1.22 (0.55-2.70)	1.68 (0.79-3.57)	1.16 (0.51-2.64)	1.69 (0.66-4.32)

Model 1: age- and survey-adjusted

Model 2: additionally adjusted for family history of stroke, physical activity, education, alcohol consumption, and smoking

Model 3: additionally adjusted for the mediators hypertension and diabetes

*based on 1717 men and 1635 women

In men, no significant association with stroke was found for any quartile of the obesity measures when compared with the first quartile after adjustment for confounders and mediating factors (model 3). However, men with a BROCA and WC over the 90^th^ percentile compared to the bottom quartile had a significantly elevated risk of ischemic stroke after adjustment for lifestyle factors (Table 4, Model 2): the significant HRs for BROCA and WC were 1.97, and 1.97, respectively (Table 4). Further adjustment for the mediating factors hypertension and history of diabetes considerably attenuated the associations, which all became non-significant (Table 4, Model 3).

On the contrary, women with a WC or a WHtR in the 4th quartile and the top 10% had a significantly increased risk for ischemic stroke even after adjustment for lifestyle factors: (WC, 4^th^ quartile: HR 3.39, 95%CI 1.53-7.54 and top 10%: HR 4.66, 95%CI 1.95 -11.17; WHtR, 4^th^ quartile: HR 2.97, 95% CI 1.37-6.42 and top 10%: HR 3.74, 95% CI 1.59-8.85, respectively; Table 4, Model 2). After further adjustment for hypertension and diabetes the association remained significant for the 4^th^ quartile and also for the top 10%.

The anthropometric measures BFMI and FFMI showed no significant association with ischemic stroke in the lifestyle- and fully adjusted models in men and women (Table 4).

Table 5 presents the RRs of ischemic stroke for the obesity measures used as continuous variables per 1 SD increase. In the age- and survey-adjusted Model all investigated anthropometric measurements (except FFMI) were significantly associated with incident ischemic stroke in both sexes. These associations remained significant even after further adjustment for family history of stroke and lifestyle factors in both sexes (except for BMI in both sexes as well as BROCA and BFMI in females; Table 5, Model 2). However, after additional adjustment for the potential mediators history of diabetes and hypertension (Model 3) all associations lost significance in men. This was not the case in women, where in the fully adjusted models the associations between WC/WHtR and stroke remained significant (RR 1.39, 95%CI 1.12-1.72 for WC and RR 1.39, 95%CI 1.12-1.73 for WHtR).

**Table 5:** Sex-specific RR of stroke for a 1-SD increase in obesity measure.

Index	Model	df	men_RR_(CI)	men_LR_test	women_RR_(CI)	women_LR_test
BMI	1	3	1.19 (1-1.42)	28	1.25 (1.01-1.53)	56.7
	2	8	1.18 (0.99-1.41)	40.9	1.22 (0.99-1.5)	65
	3	10	1.1 (0.92-1.31)	61.8	1.13 (0.91-1.4)	77.9
BROCA	1	3	1.2 (1.01-1.43)	28.5	1.23 (1-1.52)	56.3
	2	8	1.2 (1.01-1.42)	41.3	1.2 (0.98-1.48)	64.6
	3	10	1.11 (0.93-1.33)	62	1.12 (0.9-1.39)	77.7
WC	1	3	1.23 (1.04-1.46)	29.8	1.5 (1.22-1.84)	66.7
	2	8	1.21 (1.02-1.45)	42	1.48 (1.2-1.82)	74.6
	3	10	1.13 (0.94-1.35)	62.5	1.39 (1.12-1.72)	85.6
WHtR	1	3	1.28 (1.08-1.52)	32	1.49 (1.21-1.83)	65.9
	2	8	1.26 (1.06-1.51)	43.9	1.48 (1.19-1.83)	73.9
	3	10	1.17 (0.98-1.41)	63.6	1.39 (1.12-1.73)	85.2
BFMI*	1	3	1.26 (1.05-1.51)	24.8	1.3 (1.03-1.65)	45.4
	2	8	1.23 (1.03-1.48)	38.3	1.27 (1-1.6)	56.3
	3	10	1.14 (0.94-1.38)	57.6	1.18 (0.93-1.5)	69.6
FFMI*	1	3	1.16 (0.96-1.39)	21.4	1.26 (0.99-1.61)	44.3
	2	8	1.16 (0.96-1.39)	35.8	1.24 (0.98-1.58)	55.7
	3	10	1.07 (0.89-1.29)	56.4	1.12 (0.87-1.45)	68.7

*based on 1717 men and 1635 women

Model 1 adjusted for age- and survey

Model 2 additionally for family history of stroke, physical activity, education, alcohol consumption, and smoking

Model 3 additionally adjusted for the mediators hypertension and diabetes

## Discussion

### Summarizing key results

In the present prospective population-based cohort study the abdominal obesity measures WC and WHtR were independent predictors of incident ischemic stroke in women but not men aged 50 to 74 years. The general obesity measures BMI and BROCA were not associated with incident ischemic stroke in both sexes after adjustment for a number of lifestyle factors and the mediating factors diabetes and hypertension. Furthermore, neither in men nor in women, BFMI and FFMI were independent risk predictors for incident ischemic stroke.

### Abdominal obesity and incident ischemic stroke

In two German case-control studies [26, 27] abdominal obesity was a stronger risk predictor of stroke or TIA than BMI, but the significance of the association depended on vascular risk factors. Prior studies including males only found that abdominal obesity was a risk factor of stroke and that WC [7] and WHtR were better predictors [7,8,28] than BMI, which was only weakly associated with incident stroke [8, 28]. The MORGAM Study also described that indicators of abdominal adiposity especially WHtR are more strongly associated with stroke risk than BMI [29]. The Kailuan study (94,744 participants, 18–98 years old, 1,547 ischemic or haemorrhagic strokes) calculated effect estimates for BMI, WC, WHtR and waist-hip ratio (WHR) which were similar to ours [30].

In the EPIC Spanish cohort significant associations were only observed in men for WC and stroke (RR 1.95, 95%CI 1.20-3.19) and for WHtR and stroke (RR 1.58, 95%CI 1.12-2.25) [16]. The results concerning sex are opposite to ours and thus further studies are needed on this issue.

Regional abdominal fat mass measures (e.g. WC) turned out as better risk predictors for CVD than the general obesity measure BMI [8,9] most likely due to the fact that visceral fat could be regarded as endocrine organ, which is metabolically active and secretes proinflammatory cytokines [12]. Because atherosclerosis is an inflammatory disease, it could be assumed that abdominal fat could accelerate the development of atherosclerotic diseases, such as ischemic stroke. Furthermore, visceral fat is associated with cardiovascular risk factors, e.g. diabetes, hyperlipidemia, and hypertension [31]. In the present study the association between abdominal fat mass and incident ischemic stroke was partially explained by this fact, because after adjustment for the mediating factors diabetes and hypertension, the association was attenuated, particularly in men.

Sex-specific particularities with regard to the association between different obesity indices and incident stroke could be assumed. In accordance with prior population-based studies [32] our study found stronger associations between abdominal fat measures and incident ischemic stroke in women than men. Body fat distribution is sex-specific with usually an android fat accumulation in men and a gynoid accumulation in women [10]. However, with increasing age, women also tend to accumulate abdominal fat. The mechanism for the differences in body fat distribution between men and women is not clear so far, but is likely to be associated with distinct sex-differences regarding regional fatty acid storage, mobilization and oxidation [10]. It could be postulated, that particularly in women abdominal fat depots are accompanied by an excess release of inflammatory markers along with the development of atherosclerotic diseases. This assumption corresponds with the knowledge from literature that there are sex-differences regarding the association between inflammatory markers and CVD risk [13].

### General obesity and incident ischemic stroke

Previous studies investigating the association between BMI and stroke [33–35] found that elevated BMI increased the risk of (ischemic) stroke even when adjusting for age and other confounders, but the effect was largely mediated by high blood pressure, diabetes and hypercholesterolemia.

A Chinese study (74,942 women, 2403 strokes, mean follow-up 7.3 years) found a monotone dose-response relationship between BMI and incident stroke [36] and the authors of a Korean male servants' study [5] interpreted that the associations between BMI and stroke subtypes (ischemic/ haemorrhagic) were different: for ischemic stroke monotone and increasing, for haemorrhagic stroke J-shaped. In our study also an increasing relative risk for incident ischemic stroke was found with increasing BMI in the analyses using BMI as categorized variable (Table 4).

Interestingly, in the present study both general adiposity measures BMI and BROCA [37,38], had comparable relative risks regarding the prediction of ischemic stroke.

### Body fat mass index and fat free mass index and incident ischemic stroke

The results of the present population cohort extend the literature by showing for the first time that BFMI and FFMI were not associated with a significantly increased risk of ischemic stroke in 50 to 74 year old men and women. However, probably due to the low number of incident cases we found a non-significantly elevated risk estimate (HR 2.0) for the association between BFMI and stroke after adjustment for lifestyle factors and mediators in women.

In our study BIA was used to determine the BFMI and FFMI in men and women. Studies on stroke patients comparing the dual-energy x-ray absorptiometry (DEXA)- and the BIA-method to determine body composition found a good agreement between both methods (for fat free mass, kappa = 0.88, for fat mass, kappa = 0.77) [39]. Although BIA is only a crude measure of body fat and it was measured at baseline only, the findings of our study suggest, that in clinical practice, the measurement of total fat mass does not provide information for risk prediction beyond the common obesity measures. Further prospective studies on this issue are necessary to confirm or refute the present findings.

A study among older community-dwelling people between 65 and 100 years with a follow-up of 14 years which investigated the relationship of fat mass and fat free mass with ischemic stroke and coronary heart disease didn't find any effect for fat mass [17]. In a study from 2014 WHtR appeared to have the best predictive value regarding CVD and mortality; fat percentage did not add to risk prediction in middle-aged and older adults [40]. Although these findings are in accordance with the present finding, the studies are not comparable due to different age-ranges and outcomes, respectively.

### Limitations and strength

There are some limitations of the present study which must be considered. Participation in the study could influence the health behaviour during the study and change the weight and covariables. On the other side persons who decide to take part in the study are likely to be healthier than non-respondents. The stroke classification of the ICD 9 with neurologic signs is used for diagnosis with the possibility of a misclassification (detection signal bias: migraine as stroke). However, diagnoses were verified by the treating physicians, neurologists, computed tomography or magnet resonance examinations. Furthermore, information on stroke events was based on initial self-report and only the reported events were validated/confirmed. However, if a participant did not report an event during follow-up, no validation was conducted. Thus, some TIA’s or silent cerebrovascular events might have been missed. Some variables like weight, smoking status, diabetes and hypertension are time-varying, but we only examined these parameters at the baseline examination of the study. Other diseases, genetic factors, and health states, such as renal or inflammatory processes are known to influence the risk of stroke. Thus, we cannot completely exclude effects of residual confounding. Bioelectrical impedance analysis was used to assess body fat. Although this method is not the gold standard, it is a reliable method in population-based studies [41]. A further shortcoming was that BIA was performed only once. Also, a number of factors, e.g. dehydration of the participant, exercise before the examination could have influenced the measurement [42]. Different devices were used for blood pressure measurement in the two baseline surveys. Because blood pressure is not the main exposure variable in the present analysis, but only used as mediator variable, we think that if any, the bias of the results may be only marginal. In the present study the categorization into quartiles was used for analysis, so the comparison with other studies that used different cut-points is limited. The risk estimates for some of the investigated obesity indices were elevated but not significant most likely due to the partly small numbers of incident cases, particularly in women. Thus, the confidence intervals surrounding the point estimates for the found associations were wide or non-significant. Further studies including a greater number of subjects and incident cases would help determine the magnitude of the associations with greater precision.

This study has also several strengths. It includes a large number of individuals randomly drawn of a homogenous general population. The outcome events were validated by collecting information from general practitioners, hospital records, and death certificates. Data on weight and height were not self-reported but measured in a standardized way by trained personal. The follow-up time was long enough that the influencing factors could produce their impact.

### Conclusions

The abdominal obesity measures WC and WHtR were stronger risk predictors for ischemic stroke than the general obesity measurements BMI and BROCA. BFMI and FFMI did not turn out as better predictors than the common general and abdominal obesity measures. A substantial proportion of risk is mediated through the risk factors hypertension and history of diabetes. However, in women but not in men the associations between WC and WHtR and ischemic stroke were independent from these mediators. Thus, it may be of particular importance for women to prevent central obesity.
